# Expression and potential role of SNF5 in endometrial carcinoma

**DOI:** 10.1186/s12905-019-0718-1

**Published:** 2019-01-25

**Authors:** Shiying Sun, Yi Wu, Kai Zeng, Yue Zhao

**Affiliations:** 1 Department of Gynecology and Obstetrics, The Second Affiliated Hospital of Anhui Medical University, Hefei, 230601 Anhui China; 2 Department of Pathogenic Biology, Shenyang Medical College, Shenyang, 110034 Liaoning China; 3 Department of Cell Biology, Key laboratory of Cell Biology, Ministry of Public Health, and Key Laboratory of Medical Cell Biology, Ministry of Education, School of Life Sciences, China Medical University, Shenyang, 110122 Liaoning China

**Keywords:** Endometrial carcinoma, SNF5, Tumor progression, p21

## Abstract

**Background:**

SNF5 is a key protein in regulating cell proliferation and apoptosis in various cancers. However, the physiological roles of SNF5 in Endometrial carcinoma (EC), which is one of the most frequent malignancies of the female reproduction worldwide, remains unclear. This study aims to investigate the role of SNF5 and its correlation with clinicopathologic characteristics in EC.

**Methods:**

We performed immunohistochemistry to detect the SNF5 expression in 46 endometrial carcinomas and 20 normal endometrium (non-EC) specimens, as well as analyzed the correlations between SNF5 expression and clinicopathologic features of patients using a statistics software (GraphPad Prism V6.0). Western blotting had been used to confirm the protein level of SNF5 in endometrial tissues. In addition, we evaluated the correlations between SNF5 and p21 in EC.

**Results:**

The positive immunostaining rate for SNF5 in EC and non-EC specimens were 65% (30/46) and 25% (5/20) respectively, and the expression of SNF5 was dramatically increased in EC compared with the normal endometrium (*P* < 0.01). The overexpression of SNF5 was associated with the PR levels, but not with age, FIGO stage, grade, lymphatic metastasis, myometrial invasion or ER status. Knockdown of SNF5 inhibits the expression of p21.

**Conclusions:**

Our results indicate that SNF5 plays an important role of promoting oncogenesis in EC. These findings open up the possibility for various novel and effective combination therapies targeting SNF5 in the EC.

## Background

Endometrial carcinoma (EC) is one of the most common gynecological malignancy in the world [1], whose incidence has been steadily increasing for decades in most countries. In China, EC has become the second most common cancer of the female reproductive system [2, 3], with an estimated 63.4 thousand new cases in 2015 [4]. There are two types of EC, type I and type II, traditionally distinguished by its histology and clinical course. As estrogen-dependent endometrioid carcinomas, Type I usually shows the positive expression of estrogen and progesterone receptors and is highly differentiated and less aggressive. Type II represents non-endometrioid endometrial carcinoma and is highly malignancy and poor prognosis letting to worse outcomes [5]. Fortunately, most EC patients are diagnosed with endometrioid endometrial carcinomas in the initial stages of the disease and have a favorable prognosis. Nevertheless, approximately 30% of them are still diagnosed in late-stage or have occurred for metastatic, having inferior clinical outcomes [6]. Therefore, it is highly desirable to develop novel effective therapeutics for advanced EC for the sake of improvement on the patients’ outcomes.

SNF5, also known as SMARCB1, INI1 and BAF47, is a component of the SWI/SNF chromatin remodeling complex, regulating gene transcription by causing conformational changes of chromatin structure in an ATP-dependent manner [7]. Many studies have indicated that the mutated or deleted expression of SNF5 exists in various cancers including brain, lung and prostate cancers [8, 9]. However, recent studies show that SNF5 can bind to the promoter of p21 gene to modulate its transcriptional activities, promoting cell growth in tumors [10]. These findings demonstrated that SNF5 and p21 may play tumor-progressive roles in a subset of atypical cancers [11, 12], which has become a very prevalent theoretical understanding. Nevertheless, more work still needs to be done to explore the role of SNF5 in cancers.

In this study, we evaluated the potential role of SNF5 in the progression of ECs by measuring the expression levels of SNF5 in EC and normal endometrium (non-EC) tissues. Importantly, our statistical results showed a positive correlation between SNF5 and the expression of progesterone receptors (PRs) in cancer. In addition, we found that knockdown of SNF5 suppresses the p21 expression in EC cells.

## Methods

### Patients and tissue samples

A total of 86 patients who underwent hysterectomy at the Shengjing Hospital of China Medical University were included in the study. All specimens were reviewed by experts in diagnostic pathology and no patients received chemotherapy, radiotherapy, biotherapy, or any other operation before surgical treatment. 56 EC patients were performed by total hysterectomy with bilateral salpingo-oophorectomy surgery in gynecologic oncology. Information on clinicopathologic data including age, disease stage (International Federation of Gynecologists and Obstetricians (FIGO) 2009 criteria), histological grade (G1, well differentiated; G2, moderately differentiated; G3, poorly differentiated) [13], ER and PR expression and other information was extracted from pathology reports and medical records. Normal endometrial tissues were obtained from patients undergoing hysterectomies for benign uterine disease, including uterine myoma, adenomyosis, or uterine prolapse. This study was performed following the acquisition of informed consent from all patients. The experimental design of genomic and expression studies was reviewed and approved by the Medical Ethics Committee of China Medical University (2018PS495K).

Immunohistochemistry samples: Formalin-fixed paraffin-embedded tumor samples from 46 primary endometrial malignancies and 20 normal endometrial tissues were obtained from January 2008 to December 2012. EC patient characteristics are presented in Table 1. Western blotting samples: A total of 20 samples, including 10 cases of normal endometrium and 10 cases of EC were collected between August 2012 and June 2013.

**Table 1:** General characteristics of patients (*n* = 46)

Characteristics	No. (%) of patients
Age, years	
Mean [range]	59.5 [39–80]
Menopausal status	
Postmenopausal	38 (82.6)
Premenopausal	6 (13.0)
Unknown	2 (4.3)
Histology	
Endometrioid	40 (87.0)
Non-endometrioid	6 (13.0)
FIGO stage	
I	31 (67.4)
II	8 (17.4)
III	7 (15.2)
Histological grade	
G1	18 (39.1)
G2 and G3	28 (60.9)
Myometrial invasion	
≥ 1/2	15 (32.6)
< 1/2	31 (67.4)
Lymphatic metastasis	
Yes	7 (15.2)
No	39 (84.8)

*FIGO* 2009 International Federation of Gynecology and Obstetrics staging system

### Cell culture and transfection

The endometrial carcinoma cell line, Ishikawa (purchased from ATCC), was used in this study. The cells were cultured in RPMI-1640 medium (Invitrogen) containing 10% fetal bovine serum at 37 °C in a 5% CO_2_ atmosphere.

Ishikawa cells were reverse-transfected with siRNA of SNF5 or control using jetPRIME transfection reagent (Polyplus-transfection S.A.;Illkirch, France). siRNA was dissolved in jetPRIME buffer (final concentration, 25/50 nM SNF5 siRNA, 25nM control siRNA) following by adding jetPRIME reagent. After a 15-min incubation at room temperature (RT), the siRNA transfection mix was added to each well. The plate was gently rocked to mix and then returned to the incubator. After a 24-h incubation, the transfection medium was replaced by cell growth medium. Finally, the cells were harvested, and the RNA was extracted after another 24-h incubation. The siRNAs were purchased from Sigma-Aldrich and the sequences against SNF5 are as follows: forward, 5’-GAACUCACCAGAGAAGUUUdTdT-3′, reverse, 5’-AAACUUCUCUGGUGAGUUCdTdT-3’; control siRNA sequences: forward, 5’-UUCUCCGAACGUGUCACGUTT-3’, reverse, 5’-ACGUGACACGUUCGGAGAATT-3’.

### Immunohistochemistry

Immunohistochemistry (IHC) protocol was performed as the description above [14]. Briefly, paraffin-embedded tissue sections were de-paraffinized with xylene and dehydrated through graded ethanol, and then their endogenous peroxidase activity was quenched with 3% hydrogen peroxide for 30 min. Antigen retrieval used 10 mM sodium citrate buffer for 2 min. Sections were washed with PBS (phosphate-buffered saline) and blocked with goat serum for 15 min. The slides were incubated with rabbit anti-SNF5 primary antibody (A301-087A; Bethyl Laboratories) in a dilution of 1:2000 overnight at 4 °C, washed, and then were incubated for 20 min at room temperature with respective biotinylated goat anti-mouse/rabbit secondary antibody and biotinylated horseradish peroxidase complex both in the UltraSensitive™ SP (Mouse/Rabbit) IHC Kit (Maixin Bio). The sections were incubated with DAB (3,3′-diaminobenzidine tetrahydrochloride) for 5 min (Maixin Bio), washed under tap water and counterstained with hematoxylin. Finally, the slides were observed by microscopy (Olympus).

### IHC scoring

Tumor cells were scored independently for both intensity and percentage of tumor cells staining by 3 separate pathologists. The intensity of the stain was ranged from 0 to 3: 0 no staining intensity; 1 weak stained; 2 and 3 were defined as moderate and strong stained, respectively. The proportion of stained present was scored subjectively as 0 (0–5% staining), 1 (6–25% staining), 2 (26–50% staining), 3 (51–75% staining) and 4 (75–100% staining). The final score was the product of the density score and the percentage score (ranging from 0 to 12). The two grades were added together, and scores that ranged from 8 to 12 were considered as positive expression.

### Western blotting

Immunoblotting with whole-cell lysates and endometrioid tissues was performed as the description above [14]. The protein concentrations were measured by bicinchoninic acid assay (Beyotime Institute of Biotechnology). The samples containing 30 μg proteins were separated by a sodium dodecyl sulphate polyacrylamide gel electrophoresis. Proteins were transferred to polyvinylidene difluoride (PVDF) membranes and blotted on 5% non-fat milk for 1 h. Then the following primary antibodies were used: SNF5 (A301-087A; Bethyl Laboratories) in a dilution of 1:2000, p21 (CS2946; Cell Signaling Technology) in a dilution of 1:2000 and GAPDH (ab8245; Abcam) in a dilution of 1:20000. After an overnight incubation at 4 °C, the membranes were incubated with the secondary antibodies for 1 h and washed with PBST (Phosphate buffered saline with Tween-20). The secondary antibody used for anti-SNF5 was goat anti-rabbit IgG (ab6721; Abcam) in a dilution of 1:20000, anti-p21 and anti-GAPDH was goat anti-mouse IgG (ab6789; Abcam) in a dilution of 1:20000. The protein hybridization band was visualized by using the enhanced chemiluminescence reagents. The results were analyzed using ImageJ software.

### Statistical analysis

All Statistical analysis was performed using the GraphPad Prism software version 6.0 application (GraphPad Software, San Diego, CA, USA). For IHC staining, statistical differences were calculated by two-tailed analysis of Chi-square test between two or three experimental groups. For the analysis of western blotting, Kolmogorov-Smirnov test is first used to test whether data is in line with normal distribution. If the data fit normal distribution, the one-way ANOVA test is then used for the comparisons of two experimental groups; if not, the non-parametric Mann-Whitney U test is then used. A low *P*-value lesser than 0.05 (*P* < 0.05) is considered statistically significant.

## Results

### Expression and localization of SNF5 in ECs specimens

**Fig. 1:**
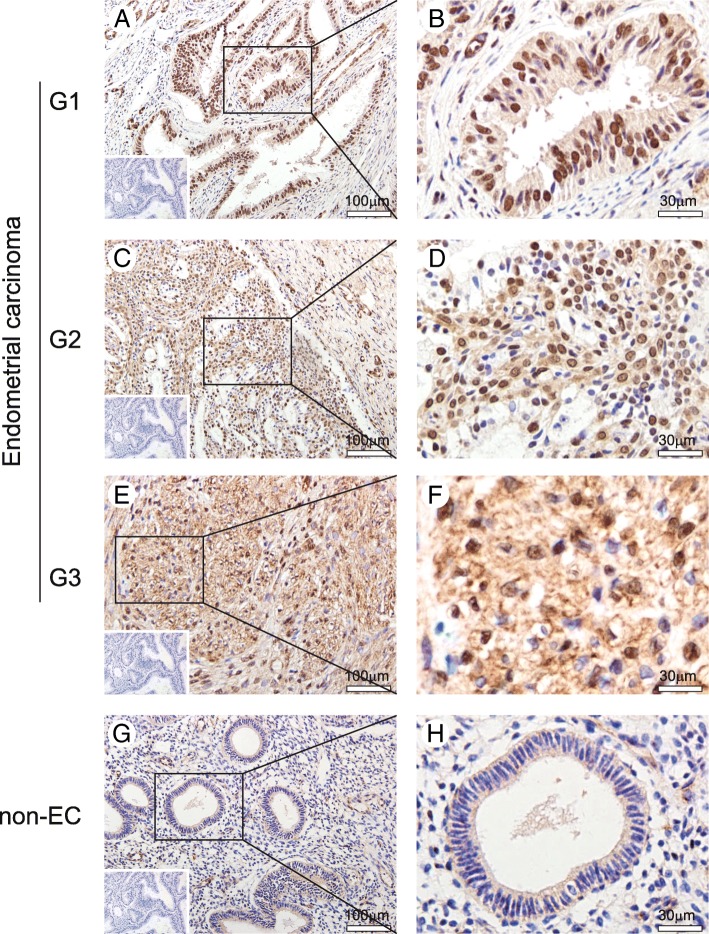
Immunohistochemical staining of SNF5 in specimens from patients with and without EC. **a** and **b** Well-differentiated (G1) EC. (**c** and **d**) Moderately differentiated (G2) EC. (**e** and **f**) Poorly differentiated (G3) EC. (**g** and **h**) Normal endometrial tissue. SNF5 is overexpressed in EC, and its staining was found mostly in nuclei. Representative images were captured at × 100 (left, scale bars: 100 μm) and × 400 (right, scale bars: 30 μm) original magnification. Insets show negative IgG controls

Aberrant SNF5 expression or mutation has been found in various tumors. However, its expression or role in ECs is still uncertain. Hence, we assessed the expression and localization of SNF5 in 46 ECs and 20 non-ECs. We found that SNF5 had strong staining in various histological grades of EC and weak staining in non-EC specimens (Fig. 1). In addition, the positive staining of SNF5 was mainly observed in the nucleus. Semiquantitative analysis showed that over half of the EC patients (30/46, 65%) has a positive expression of SNF5, whereas only a quarter of the normal endometrium specimens present positive expression, indicating that SNF5 has a higher staining level in ECs specimens than that in non-ECs (*P* = 0.0026, shown in Table 2).

**Table 2:** SNF5 expression in each group

Groups	Samples (n)	SNF5 expression		*P*-value
		Positive	Negative	
EC	46	30	16	0.0026**
non-EC	20	5	15	

** *P* < 0.01

### Statistics of SNF5 expression and clinicopathological characteristics

Based on the finding of the SNF5 overexpression of in a large proportion of ECs, the clinicopathological characteristics of SNF5-positive and SNF5-negative patients after review are summarized in Table 3. Patients with SNF5-positive tumors present a stronger expression of PR compared to those with SNF5-negative tumors. Such result indicates that increased expression of SNF5 is notably associated with the PR expression (*P* = 0.0243), but there is no significant correlation between the intensity of SNF5 expression and age, pathologic subtype, FIGO stage, histological grade, lymphatic metastasis, myometrial invasion or ER expression.

**Table 3:** Correlation between SNF5 expression and different clinicopathologic features in ECs

Characteristics	Samples (n)	SNF5 expression		*P*-value
		Positive	Negative	
Ages				
≥ 50	40	25	15	0.318
< 50	6	5	1	
Pathologic subtype				
Endometrioid	40	28	12	0.079
Non-endometrioid	6	2	4	
FIGO stage				
I	31	20	11	0.761
II	8	6	2	
III	7	4	3	
Histological grade				
G1	18	14	4	0.152
G2 and G3	28	16	12	
Myometrial invasion				
≥ 1/2	15	12	3	0.626
< 1/2	31	18	13	
Lymphatic metastasis				
Yes	7	4	3	0.143
No	39	26	13	
ER expression				
positive	29	19	10	0.956
negative	17	11	6	
PR expression				
positive	31	23	8	0.024*
negative	15	6	9	

*FIGO* 2009 International Federation of Gynecology and Obstetrics staging system

*P < 0.05

### Protein expression of SNF5 in EC tissues

**Fig. 2:**
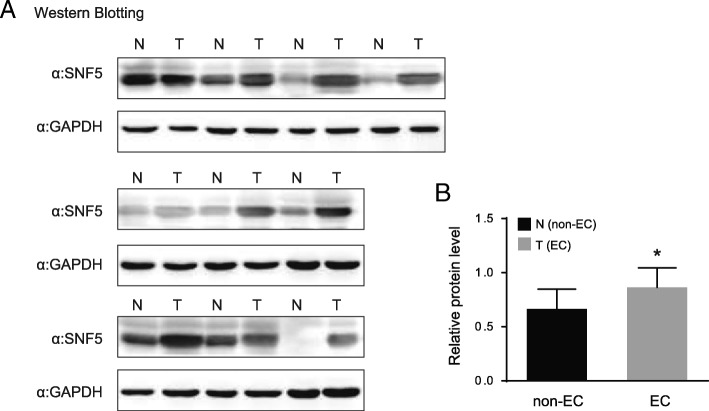
Protein expression of SNF5 in 20 fresh tissue specimens from patients with or without EC. **a** Western blotting assay using an antibody against SNF5, and samples were examined in all. **b** Expression was higher in EC tissue specimens than in non-EC tissue specimens. N = control tissue specimen (non-EC); T = tumor tissue specimen (EC). Protein bands were analysed by one-way ANOVA test with GraphPad 6.0 software. Data are expressed as mean ± SD of three independent experiments, *P < 0.05

In order to further confirm the results from histologic specimens in EC, we detected the protein levels of SNF5 in 10 EC and non-EC tissue lysates respectively. Western blotting results indicate that the expression level of SNF5 is stronger in EC than that in non-EC (Fig. 2a). By comparing two groups of patients, a statistically significant difference of SNF5 level (*P* < 0.05) was found (Fig. 2b).

### Analysis of the correlation between SNF5 and p21 in Ishikawa cells

**Fig. 3:**
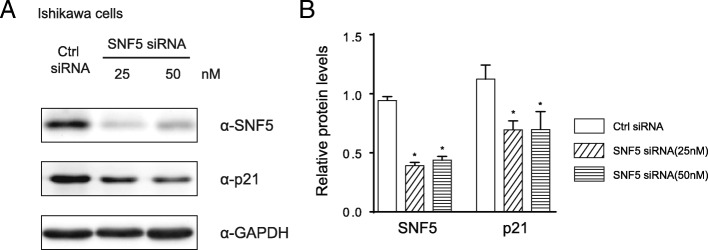
Western blotting analysis. Ishikawa cells were collected after transfection with two concentration siRNA (25 or 50 nM) against SNF5 or control siRNA. (**a**) Precipitated proteins were measured by western blotting using antibodies targeting SNF5 or p21. GAPDH was used as an internal control. (**b**) The western blotting results were analysed by Mann-Whitney test using GraphPad 6.0 software. Data are shown as the mean ± SD of three independent experiments, *P < 0.05

Previous studies suggested that SNF5 could affect cell proliferation via regulating p21 transcription in malignant rhabdoid tumors [15]. Therefore, we further evaluated whether the p21 expression is associated with SNF5 in EC using the Ishikawa cell line. The Ishikawa cell line is an excellent model for type I endometrial cancer which over 80% EC patients are diagnosed with, and thus Ishikawa cell line is the most widely used in EC studies compared with EC cell lines at other stages [16]. To this end, we transfected 10, 25 and 50 nM concentrations respectively to Ishikawa cells in the pre-experiment according to the protocol of the transfection reagent and found that 25 nM is the optimal concentration of siRNA against SNF5 while 50 nM is the near optimal concentration. A significantly reduced expression of p21 was observed with SNF5 silence by western blotting (Fig. 3). This result indicates that knockdown of SNF5 down-regulates p21 levels in EC cells. Based on above, the data from clinical samples combined with results from Ishikawa cells give strong evidence that SNF5 plays an oncogene role in EC by regulating p21 activation.

## Discussion

In general, EC patients are mostly in the class of type I, which mostly belongs to endometrial adenocarcinoma, is associated with unopposed estrogen stimulation, and often preceded by endometrial hyperplasia not spreading beyond the uterus [6, 17]. However, it is unreliable to distinguish the biological behavior of carcinomas just depended on the pathological type. The cases of endometrial adenocarcinoma are not entirely consistent with the features of type I EC. For instance, the patients with a high malignant degree such as myometrial invasion and chemotherapy resistance are considerably common in EC cases [18, 19]. Therefore, it is essential to understand its molecular biological characteristics for the process determination of tumor and malignancy, and the appropriate option of individualized treatment. In recent years, there has been an increasing awareness that epigenetic regulation plays a crucial role in the modulation of gene expression and tumorigenesis, such as DNA methylation, histone modification and chromatin remodeling [7, 20]. In the cases of SNF5, which is a core subunit of SWI/SNF complex, it utilizes the energy of ATP hydrolysis to remodel chromatin and regulate transcription to exert its functions in oncogenesis [21, 22]. In spite of its potent role in many tumors, the expression and function of SNF5 in EC is still unknown.

In this study, we focused on the role and clinical relevance of SNF5 in EC and non-EC. SNF5 proteins are expressed in both EC and non-EC specimens in immunostaining. However, the positive expression of the SNF5 protein in EC specimens is at 65.2%, remarkably higher than that in non-EC specimens (25%, *P* = 0.0026). Moreover, the SNF5 expression in western blotting is also higher in EC than that in non-EC tissue samples (*P* < 0.05). These results give strong evidence that SNF5 expression was significantly increased in EC compared with that in non-EC specimens. In addition, the location of SNF5 protein was mainly observed in the nucleus, consistent with previous reports of nuclear localization of SNF5 in malignant rhabdoid tumors [23]. The statistical analysis shows that the SNF5 staining status was correlated with the expression of PR (*P* < 0.05), indicating SNF5 play an important role in PR-mediated signal transduction pathways in cancers. In contrast, no relationship was found between SNF5 expression and other features including age, pathologic subtype, FIGO stage, tumor grade, lymphatic metastasis, myometrial invasion, and ER status. There are more works need to do in future, including verifying the tumorigenesis mechanism and clinical implications of SNF5 in EC, to further improve the insight into the role of SNF5 in EC.

In previous studies, the biallelic inactivation of the SNF5 gene is associated with the occurrence of malignant rhabdoid tumors, and the absence of SNF5 expression is detected in synovial sarcoma and epithelioid sarcomas where SNF5 is considered as a tumor suppressor [23–25]. However, other studies show that the knockdown of SNF5 in tumor cells leads to a decreased expression of p53 and its target gene p21, as well as cell-cycle arrest in G1 and apoptosis [8, 26, 27]. In this study, the increased expression of SNF5 in EC was observed, consistent with the latter findings of a high SNF5 level in cancers. Besides, we also found that silence of SNF5 expression induces a lower level of p21 in EC cells, further confirming that the mechanism of SNF5 carcinogenesis in EC is related to the oncogenic activities of p21 in tumors. Actually, SNF5 has been reported as a chromatin remodeling factor which is recruited to the p21 promoter and modulates the transcriptional activity of p21 to facilitate the cell proliferation [15]. Moreover, it has been confirmed that p21 is colocalized with the proliferation marker Ki67 and overexpresses in a large number of human cancers including lung, prostate, breast, cervical and precancerous cancers [12, 28]. Therefore, our results, together with many previous findings, indicate that SNF5 is a tumor promoter rather than a tumor suppressor and facilitate cancer process in EC.

## Conclusion

In summary, our studies suggest that SNF5 plays a critical role as a tumor promoter by regulating the levels of p21 in endometrial carcinoma. Our statistic analysis reveals a positive correlation between SNF5 expression and the PR expression. These results provide important information regarding the role of SNF5 in EC and pave the way for the early prognosis and the targeted therapy of EC.

## Abbreviations

ANOVA: Analysis of Variance
ATCC: American type culture collection
EC: Endometrial carcinoma
ER: Estrogen receptor
FIGO: International federation of gynecology and obstetrics
IHC: Immunohistochemistry
PR: Progesterone receptor
